# Bis{1-[(*E*)-*o*-tolyl­diazen­yl]-2-naphtho­l­ato}copper(II)

**DOI:** 10.1107/S1600536810037888

**Published:** 2010-09-30

**Authors:** Wan-Ju Tai, Chi-Huan Li, Chen-Yu Li, Bao-Tsan Ko

**Affiliations:** a Department of Chemistry, Chung Yuan Christian University, Chung-Li 320, Taiwan

## Abstract

In the title complex, [Cu(C_17_H_13_N_2_O)_2_], the Cu^II^ atom is tetra­coordinated by two N atoms and two O atoms from two bidentate 1-[(*E*)-*o*-tolyl­diazen­yl]-2-naphtho­late ligands, forming a slightly distorted square-planar environment. The two N atoms and two O atoms around the Cu^II^ atom are *trans* to each other, with an O—Cu—O bond angle of 177.00 (9)° and an N—Cu—N bond angle of 165.63 (10)°. The average distances between the Cu^II^ atom and the coordinated O and N atoms are 1.905 (2) and 1.995 (2)Å, respectively.

## Related literature

For background to 1-phenyl­azo-2-naphtol derivatives, see: Shen *et al.* (2003 ▶); Lin *et al.* (2010 ▶). For related structures: see: Lin *et al.* (2008 ▶).
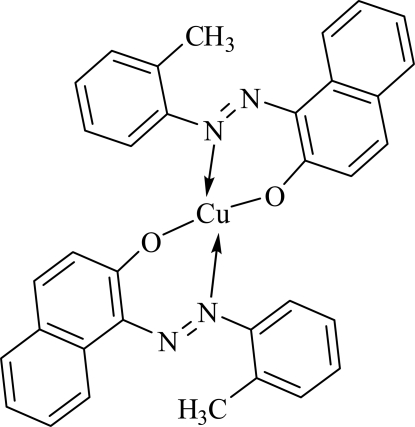


## Experimental

### 

#### Crystal data

[Cu(C_17_H_13_N_2_O)_2_]
*M* *_r_* = 586.14
Monoclinic, 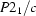

*a* = 12.9777 (2) Å
*b* = 15.2771 (3) Å
*c* = 15.1674 (2) Å
β = 114.514 (1)°
*V* = 2736.05 (8) Å^3^
*Z* = 4
Mo *K*α radiation
μ = 0.84 mm^−1^
*T* = 296 K
0.18 × 0.12 × 0.10 mm

#### Data collection

Bruker APEXII CCD diffractometer
Absorption correction: multi-scan (*SADABS*; Bruker, 2008 ▶) *T*_min_ = 0.864, *T*_max_ = 0.921
25896 measured reflections
6767 independent reflections
3353 reflections with *I* > 2σ(*I*)
*R*_int_ = 0.067

#### Refinement

*R*[*F* ^2^ > 2σ(*F* ^2^)] = 0.051
*wR*(*F* ^2^) = 0.117
*S* = 1.02
6767 reflections
372 parameters
H-atom parameters constrained
Δρ_max_ = 0.30 e Å^−3^
Δρ_min_ = −0.43 e Å^−3^

### 

Data collection: *APEX2* (Bruker, 2008 ▶); cell refinement: *SAINT-Plus* (Bruker, 2008 ▶); data reduction: *SAINT-Plus*; program(s) used to solve structure: *SHELXS97* (Sheldrick, 2008 ▶); program(s) used to refine structure: *SHELXL97* (Sheldrick, 2008 ▶); molecular graphics: *SHELXTL* (Sheldrick, 2008 ▶); software used to prepare material for publication: *SHELXTL*.

## Supplementary Material

Crystal structure: contains datablocks I, global. DOI: 10.1107/S1600536810037888/rk2236sup1.cif

Structure factors: contains datablocks I. DOI: 10.1107/S1600536810037888/rk2236Isup2.hkl

Additional supplementary materials:  crystallographic information; 3D view; checkCIF report

## Figures and Tables

**Fig. 1.**
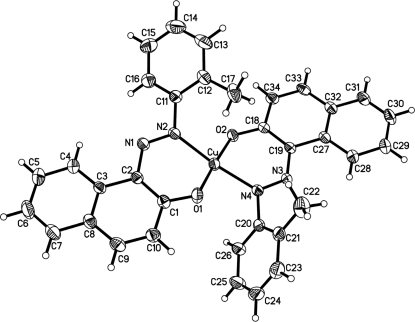
A view of the molecular structure of title compound with the atom numbering scheme. Displacement ellipsoids are drawn at the 30% probability level. H atoms are presented as a small spheres of arbitrary radius.

**Fig. 2.**
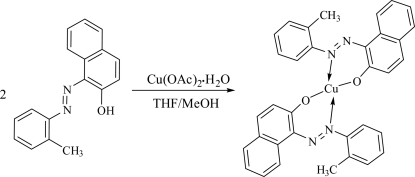
The synthetic procedure of the title compound.

## supplementary crystallographic information

### Comment

Recently, the 1-phenylazo-2-naphtol (*PAN*-H) derivatives attract our attentions because the phenylazo-naphtolate group can provide the *N,O*-bidentate chelation to stabilize the transition metal or main group metal complexes. Therefore, our group is interested in the synthesis and preparation of metal complexes bearing such a ligand of this kind. For instance, our group has successfully synthesized and structural characterized the Pd(II) complex with *N,O*-bidentate phenylazo-naphtolate ligands (Lin *et al.*, 2010). In addition, Shen *et al.*, (2003) reported that Zn, Cu, and Co complexes supported by *N,N,O,O*-tetradentate binaphthyldiamino salen ligands have been demonstrated effectively to catalyze epoxides and CO_2_ coupling reactions to achieve five-membered ring cyclic carbonates. In order to develop various metal systems originated from *PAN* derivatives and to investigate the catalytic behavior of these complexes, we report herein the synthesis and crystal structure of the title compound, (I), a potential catalyst for CO_2_/epoxide coupling reactions (Scheme 1).

The solid structure of (I) reveals a monomeric Cu^II^ complex (Fig. 1) containing two six-membered rings coordinated from these two *N,O*-bidentate phenylazo-naphtolate ligands. The geometry around Cu atom is tetra-coordinated with a slight distorted square planar environment and two nitrogen atoms and two oxygen atoms are almost coplanar in which the sums of bond angles around Cu center are 359.81 (9)°. The two N atoms and two O atoms around Cu^II^ atom are *trans*- to each other with O1–Cu–O2 bond angle of 177.00 (9)° and N2–Cu–N4 bond angle of 165.63 (10)°. The distances between the Cu atom and O1, O2, N2 and N4 are 1.887 (2), 1.9234 (19), 2.001 (2) and 1.988 (2)Å, respectively. These average bond distances are similar to those found in the crystal structure of (5-methoxy-2-{1-[2-(dimethylamino)ethylimino]benzyl}phenolato)copper(II) acetate (Lin *et al.*, 2008).

### Experimental

The title compound (I) was synthesized by the following procedures (Fig. 2): (*E*)-1-(*o*-tolyldiazenyl)naphthalen-2-ol (0.52 g, 2.0 mmol) and Cu(O*Ac*)_2_^.^H_2_O (0.20 g, 1.0 mmol) was stirred at 298 K in the mixture of *THF*/*Me*OH (10/10 ml) for 24 h. Volatile materials were removed under vacuum and the residue was washed twice from hexane solution to give dark brown solids. The resulting solids were crystallized from CH_2_Cl_2_/Hexane (1:5) solution to yield brown crystals.

### Refinement

The H atoms were placed in idealized positions and constrained to ride on their parent atoms, with C–H = 0.93Å with *U_iso_*(H) = 1.2*U*_eq_(C) for aromatic hydrogen; C–H = 0.96Å with *U*_iso_(H) = 1.5*U*_eq_(C) for CH_3_-group.

### Crystal data

**Table:**

[Cu(C_17_H_13_N_2_O)_2_]	*F*(000) = 1212
*M**_r_* = 586.14	*D*_x_ = 1.423 Mg m^−^^3^
Monoclinic, *P*2_1_/*c*	Mo *K*α radiation, λ = 0.71073 Å
Hall symbol: -P 2ybc	Cell parameters from 6095 reflections
*a* = 12.9777 (2) Å	θ = 2.2–23.7°
*b* = 15.2771 (3) Å	µ = 0.84 mm^−^^1^
*c* = 15.1674 (2) Å	*T* = 296 K
β = 114.514 (1)°	Block, brown
*V* = 2736.05 (8) Å^3^	0.18 × 0.12 × 0.10 mm
*Z* = 4	

### Data collection

**Table:**

Bruker APEXII CCD diffractometer	6767 independent reflections
Radiation source: fine-focus sealed tube	3353 reflections with *I* > 2σ(*I*)
graphite	*R*_int_ = 0.067
Detector resolution: 8.3333 pixels mm^-1^	θ_max_ = 28.4°, θ_min_ = 2.0°
φ and ω scans	*h* = −17→14
Absorption correction: multi-scan (*SADABS*; Bruker, 2008)	*k* = −16→20
*T*_min_ = 0.864, *T*_max_ = 0.921	*l* = −20→19
25896 measured reflections	

### Refinement

**Table:**

Refinement on *F*^2^	Primary atom site location: structure-invariant direct methods
Least-squares matrix: full	Secondary atom site location: difference Fourier map
*R*[*F*^2^ > 2σ(*F*^2^)] = 0.051	Hydrogen site location: inferred from neighbouring sites
*wR*(*F*^2^) = 0.117	H-atom parameters constrained
*S* = 1.02	*w* = 1/[σ^2^(*F*_o_^2^) + (0.043*P*)^2^] where *P* = (*F*_o_^2^ + 2*F*_c_^2^)/3
6767 reflections	(Δ/σ)_max_ = 0.001
372 parameters	Δρ_max_ = 0.30 e Å^−^^3^
0 restraints	Δρ_min_ = −0.43 e Å^−^^3^

### Special details

**Table:**

Geometry. All s.u.'s (except the s.u. in the dihedral angle between two l.s. planes) are estimated using the full covariance matrix. The cell s.u.'s are taken into account individually in the estimation of s.u.'s in distances, angles and torsion angles; correlations between s.u.'s in cell parameters are only used when they are defined by crystal symmetry. An approximate (isotropic) treatment of cell s.u.'s is used for estimating s.u.'s involving l.s. planes.
Refinement. Refinement of *F*^2^ against ALL reflections. The weighted *R*-factor *wR* and goodness of fit *S* are based on *F*^2^, conventional *R*-factors *R* are based on *F*, with *F* set to zero for negative *F*^2^. The threshold expression of *F*^2^ > σ(*F*^2^) is used only for calculating\ *R*-factors(gt) *etc*. and is not relevant to the choice of reflections for refinement. *R*-factors based on *F*^2^ are statistically about twice as large as those based on *F*, and *R*-factors based on ALL data will be even larger.

### Fractional atomic coordinates and isotropic or equivalent isotropic displacement parameters (Å^2^)

**Table:**

	*x*	*y*	*z*	*U*_iso_*/*U*_eq_	
Cu	0.42422 (3)	0.43237 (2)	0.40521 (3)	0.04113 (14)	
O1	0.26886 (17)	0.40571 (14)	0.33857 (14)	0.0500 (6)	
O2	0.58193 (16)	0.46328 (13)	0.46746 (13)	0.0421 (5)	
N1	0.3598 (2)	0.29328 (16)	0.50680 (16)	0.0398 (6)	
N2	0.4367 (2)	0.32243 (16)	0.48074 (16)	0.0404 (6)	
N3	0.4821 (2)	0.54485 (15)	0.27524 (17)	0.0386 (6)	
N4	0.40695 (19)	0.52061 (16)	0.30368 (17)	0.0387 (6)	
C1	0.2076 (3)	0.3729 (2)	0.3789 (2)	0.0399 (8)	
C2	0.2525 (2)	0.32424 (19)	0.4659 (2)	0.0366 (7)	
C3	0.1783 (2)	0.29426 (19)	0.5098 (2)	0.0394 (7)	
C4	0.2194 (3)	0.2519 (2)	0.5991 (2)	0.0566 (9)	
H4	0.2966	0.2410	0.6319	0.068*	
C5	0.1471 (3)	0.2264 (3)	0.6388 (3)	0.0719 (11)	
H5	0.1756	0.1973	0.6980	0.086*	
C6	0.0324 (3)	0.2431 (3)	0.5925 (3)	0.0720 (11)	
H6	−0.0156	0.2259	0.6209	0.086*	
C7	−0.0103 (3)	0.2845 (2)	0.5058 (3)	0.0608 (10)	
H7	−0.0876	0.2958	0.4751	0.073*	
C8	0.0612 (3)	0.3105 (2)	0.4618 (2)	0.0465 (8)	
C9	0.0187 (3)	0.3526 (2)	0.3700 (2)	0.0522 (9)	
H9	−0.0592	0.3596	0.3362	0.063*	
C10	0.0871 (3)	0.3826 (2)	0.3304 (2)	0.0484 (8)	
H10	0.0557	0.4101	0.2703	0.058*	
C11	0.5382 (3)	0.2696 (2)	0.5204 (2)	0.0429 (8)	
C12	0.5964 (3)	0.2492 (2)	0.4643 (2)	0.0500 (8)	
C13	0.6926 (3)	0.1982 (3)	0.5060 (3)	0.0721 (11)	
H13	0.7339	0.1845	0.4704	0.086*	
C14	0.7285 (3)	0.1672 (3)	0.5995 (4)	0.0857 (13)	
H14	0.7938	0.1332	0.6260	0.103*	
C15	0.6700 (3)	0.1856 (3)	0.6532 (3)	0.0793 (12)	
H15	0.6933	0.1627	0.7153	0.095*	
C16	0.5752 (3)	0.2386 (2)	0.6149 (2)	0.0608 (10)	
H16	0.5363	0.2536	0.6523	0.073*	
C17	0.5536 (3)	0.2733 (2)	0.3584 (2)	0.0692 (11)	
H17A	0.5837	0.3293	0.3524	0.104*	
H17B	0.4724	0.2761	0.3303	0.104*	
H17C	0.5776	0.2299	0.3252	0.104*	
C18	0.6409 (2)	0.48514 (18)	0.4187 (2)	0.0349 (7)	
C19	0.5933 (2)	0.52256 (19)	0.3255 (2)	0.0355 (7)	
C20	0.2942 (3)	0.5505 (2)	0.2400 (2)	0.0416 (8)	
C21	0.2460 (3)	0.5269 (2)	0.1430 (2)	0.0484 (8)	
C22	0.3046 (3)	0.4726 (3)	0.0960 (3)	0.0756 (12)	
H22A	0.3552	0.5089	0.0804	0.113*	
H22B	0.2494	0.4470	0.0378	0.113*	
H22C	0.3469	0.4271	0.1396	0.113*	
C23	0.1347 (3)	0.5547 (3)	0.0899 (3)	0.0699 (11)	
H23	0.0990	0.5408	0.0244	0.084*	
C24	0.0770 (3)	0.6014 (3)	0.1308 (4)	0.0765 (13)	
H24	0.0029	0.6187	0.0930	0.092*	
C25	0.1259 (3)	0.6232 (3)	0.2259 (3)	0.0722 (11)	
H25	0.0859	0.6558	0.2532	0.087*	
C26	0.2350 (3)	0.5969 (2)	0.2819 (3)	0.0548 (9)	
H26	0.2687	0.6103	0.3476	0.066*	
C27	0.6647 (3)	0.54545 (19)	0.2761 (2)	0.0398 (8)	
C28	0.6209 (3)	0.5812 (2)	0.1826 (2)	0.0510 (9)	
H28	0.5432	0.5897	0.1495	0.061*	
C29	0.6913 (3)	0.6037 (2)	0.1393 (3)	0.0634 (10)	
H29	0.6611	0.6282	0.0776	0.076*	
C30	0.8071 (3)	0.5902 (2)	0.1869 (3)	0.0680 (11)	
H30	0.8543	0.6058	0.1572	0.082*	
C31	0.8512 (3)	0.5544 (2)	0.2760 (3)	0.0581 (10)	
H31	0.9290	0.5451	0.3071	0.070*	
C32	0.7820 (3)	0.5310 (2)	0.3228 (2)	0.0438 (8)	
C33	0.8260 (3)	0.4931 (2)	0.4167 (2)	0.0476 (8)	
H33	0.9035	0.4833	0.4481	0.057*	
C34	0.7596 (2)	0.4708 (2)	0.4623 (2)	0.0439 (8)	
H34	0.7925	0.4454	0.5236	0.053*	

### Atomic displacement parameters (Å^2^)

**Table:**

	*U*^11^	*U*^22^	*U*^33^	*U*^12^	*U*^13^	*U*^23^
Cu	0.0344 (2)	0.0535 (3)	0.0367 (2)	−0.00050 (19)	0.01588 (18)	0.00637 (19)
O1	0.0418 (14)	0.0691 (16)	0.0385 (12)	−0.0103 (11)	0.0159 (11)	0.0092 (11)
O2	0.0333 (12)	0.0610 (14)	0.0338 (11)	0.0007 (10)	0.0156 (10)	0.0040 (10)
N1	0.0353 (16)	0.0466 (16)	0.0395 (14)	0.0009 (13)	0.0173 (13)	−0.0001 (12)
N2	0.0338 (15)	0.0501 (17)	0.0399 (15)	0.0051 (12)	0.0179 (13)	0.0030 (12)
N3	0.0330 (16)	0.0475 (16)	0.0389 (14)	0.0022 (12)	0.0186 (13)	0.0060 (12)
N4	0.0299 (15)	0.0493 (16)	0.0371 (15)	0.0013 (12)	0.0141 (13)	0.0047 (12)
C1	0.037 (2)	0.044 (2)	0.0384 (18)	−0.0052 (15)	0.0157 (16)	−0.0051 (15)
C2	0.0329 (19)	0.0407 (18)	0.0362 (17)	−0.0014 (14)	0.0142 (15)	−0.0021 (14)
C3	0.038 (2)	0.0390 (18)	0.0466 (19)	0.0029 (14)	0.0235 (16)	−0.0009 (15)
C4	0.058 (2)	0.062 (2)	0.061 (2)	0.0086 (18)	0.037 (2)	0.0172 (19)
C5	0.075 (3)	0.083 (3)	0.070 (3)	0.001 (2)	0.043 (2)	0.023 (2)
C6	0.070 (3)	0.081 (3)	0.089 (3)	−0.009 (2)	0.056 (3)	0.005 (2)
C7	0.048 (2)	0.067 (3)	0.077 (3)	−0.0016 (19)	0.035 (2)	−0.003 (2)
C8	0.049 (2)	0.045 (2)	0.055 (2)	−0.0040 (16)	0.0300 (18)	−0.0076 (16)
C9	0.035 (2)	0.063 (2)	0.054 (2)	−0.0039 (17)	0.0144 (18)	−0.0071 (18)
C10	0.038 (2)	0.061 (2)	0.0408 (19)	−0.0004 (16)	0.0106 (17)	−0.0003 (16)
C11	0.040 (2)	0.044 (2)	0.046 (2)	0.0008 (15)	0.0189 (16)	0.0008 (15)
C12	0.049 (2)	0.050 (2)	0.060 (2)	−0.0052 (17)	0.0315 (18)	−0.0105 (17)
C13	0.057 (3)	0.069 (3)	0.104 (3)	0.007 (2)	0.047 (3)	−0.014 (3)
C14	0.059 (3)	0.078 (3)	0.113 (4)	0.020 (2)	0.029 (3)	0.008 (3)
C15	0.063 (3)	0.088 (3)	0.077 (3)	0.019 (2)	0.019 (2)	0.028 (2)
C16	0.053 (2)	0.074 (3)	0.056 (2)	0.008 (2)	0.0230 (19)	0.011 (2)
C17	0.091 (3)	0.065 (3)	0.077 (3)	−0.010 (2)	0.060 (2)	−0.015 (2)
C18	0.0309 (18)	0.0362 (18)	0.0389 (18)	0.0007 (14)	0.0157 (15)	−0.0035 (14)
C19	0.0293 (18)	0.0410 (18)	0.0382 (18)	−0.0002 (14)	0.0161 (15)	−0.0030 (14)
C20	0.0328 (19)	0.045 (2)	0.049 (2)	−0.0027 (15)	0.0190 (17)	0.0090 (16)
C21	0.034 (2)	0.062 (2)	0.047 (2)	−0.0109 (16)	0.0148 (17)	0.0090 (18)
C22	0.080 (3)	0.090 (3)	0.056 (2)	−0.023 (2)	0.027 (2)	−0.014 (2)
C23	0.042 (2)	0.099 (3)	0.054 (2)	−0.018 (2)	0.006 (2)	0.020 (2)
C24	0.034 (2)	0.097 (3)	0.088 (3)	0.003 (2)	0.015 (2)	0.039 (3)
C25	0.049 (3)	0.073 (3)	0.104 (3)	0.013 (2)	0.041 (3)	0.017 (3)
C26	0.040 (2)	0.060 (2)	0.066 (2)	0.0055 (17)	0.0244 (19)	0.0097 (19)
C27	0.037 (2)	0.0416 (19)	0.0468 (19)	0.0008 (14)	0.0232 (16)	−0.0020 (15)
C28	0.046 (2)	0.062 (2)	0.053 (2)	0.0037 (17)	0.0276 (18)	0.0099 (17)
C29	0.068 (3)	0.074 (3)	0.066 (2)	0.009 (2)	0.047 (2)	0.017 (2)
C30	0.064 (3)	0.080 (3)	0.086 (3)	0.001 (2)	0.057 (2)	0.010 (2)
C31	0.042 (2)	0.069 (3)	0.074 (3)	0.0005 (18)	0.034 (2)	0.005 (2)
C32	0.039 (2)	0.0449 (19)	0.053 (2)	−0.0031 (15)	0.0252 (17)	−0.0056 (16)
C33	0.0248 (18)	0.059 (2)	0.057 (2)	−0.0002 (15)	0.0153 (17)	−0.0090 (18)
C34	0.032 (2)	0.057 (2)	0.0364 (18)	0.0049 (15)	0.0084 (15)	−0.0021 (15)

### Geometric parameters (Å, °)

**Table:**

Cu—O1	1.887 (2)	C15—C16	1.384 (5)
Cu—O2	1.9234 (19)	C15—H15	0.9300
Cu—N4	1.988 (2)	C16—H16	0.9300
Cu—N2	2.001 (2)	C17—H17A	0.9600
O1—C1	1.288 (3)	C17—H17B	0.9600
O2—C18	1.309 (3)	C17—H17C	0.9600
N1—N2	1.295 (3)	C18—C19	1.408 (4)
N1—C2	1.353 (3)	C18—C34	1.418 (4)
N2—C11	1.446 (4)	C19—C27	1.455 (4)
N3—N4	1.273 (3)	C20—C26	1.379 (4)
N3—C19	1.367 (3)	C20—C21	1.387 (4)
N4—C20	1.452 (4)	C21—C23	1.397 (5)
C1—C2	1.412 (4)	C21—C22	1.490 (5)
C1—C10	1.434 (4)	C22—H22A	0.9600
C2—C3	1.454 (4)	C22—H22B	0.9600
C3—C4	1.391 (4)	C22—H22C	0.9600
C3—C8	1.408 (4)	C23—C24	1.356 (6)
C4—C5	1.365 (4)	C23—H23	0.9300
C4—H4	0.9300	C24—C25	1.355 (5)
C5—C6	1.381 (5)	C24—H24	0.9300
C5—H5	0.9300	C25—C26	1.375 (5)
C6—C7	1.352 (5)	C25—H25	0.9300
C6—H6	0.9300	C26—H26	0.9300
C7—C8	1.407 (4)	C27—C28	1.401 (4)
C7—H7	0.9300	C27—C32	1.405 (4)
C8—C9	1.421 (4)	C28—C29	1.372 (4)
C9—C10	1.342 (4)	C28—H28	0.9300
C9—H9	0.9300	C29—C30	1.387 (5)
C10—H10	0.9300	C29—H29	0.9300
C11—C12	1.387 (4)	C30—C31	1.346 (5)
C11—C16	1.392 (4)	C30—H30	0.9300
C12—C13	1.382 (5)	C31—C32	1.403 (4)
C12—C17	1.512 (4)	C31—H31	0.9300
C13—C14	1.380 (5)	C32—C33	1.419 (4)
C13—H13	0.9300	C33—C34	1.354 (4)
C14—C15	1.354 (5)	C33—H33	0.9300
C14—H14	0.9300	C34—H34	0.9300
			
O1—Cu—O2	177.00 (9)	C15—C16—H16	120.1
O1—Cu—N4	88.82 (9)	C11—C16—H16	120.1
O2—Cu—N4	88.21 (9)	C12—C17—H17A	109.5
O1—Cu—N2	86.57 (9)	C12—C17—H17B	109.5
O2—Cu—N2	96.21 (9)	H17A—C17—H17B	109.5
N4—Cu—N2	165.63 (10)	C12—C17—H17C	109.5
C1—O1—Cu	124.20 (19)	H17A—C17—H17C	109.5
C18—O2—Cu	122.56 (18)	H17B—C17—H17C	109.5
N2—N1—C2	121.1 (2)	O2—C18—C19	123.7 (3)
N1—N2—C11	111.4 (2)	O2—C18—C34	118.3 (3)
N1—N2—Cu	125.01 (19)	C19—C18—C34	118.1 (3)
C11—N2—Cu	123.40 (19)	N3—C19—C18	125.7 (3)
N4—N3—C19	121.2 (2)	N3—C19—C27	113.8 (3)
N3—N4—C20	112.7 (2)	C18—C19—C27	120.4 (3)
N3—N4—Cu	127.11 (19)	C26—C20—C21	122.0 (3)
C20—N4—Cu	119.11 (18)	C26—C20—N4	117.1 (3)
O1—C1—C2	123.5 (3)	C21—C20—N4	120.7 (3)
O1—C1—C10	118.2 (3)	C20—C21—C23	115.8 (3)
C2—C1—C10	118.2 (3)	C20—C21—C22	123.8 (3)
N1—C2—C1	124.0 (3)	C23—C21—C22	120.3 (3)
N1—C2—C3	115.5 (3)	C21—C22—H22A	109.5
C1—C2—C3	120.0 (3)	C21—C22—H22B	109.5
C4—C3—C8	118.7 (3)	H22A—C22—H22B	109.5
C4—C3—C2	122.2 (3)	C21—C22—H22C	109.5
C8—C3—C2	119.0 (3)	H22A—C22—H22C	109.5
C5—C4—C3	120.4 (3)	H22B—C22—H22C	109.5
C5—C4—H4	119.8	C24—C23—C21	122.1 (4)
C3—C4—H4	119.8	C24—C23—H23	118.9
C4—C5—C6	121.0 (4)	C21—C23—H23	118.9
C4—C5—H5	119.5	C25—C24—C23	120.9 (4)
C6—C5—H5	119.5	C25—C24—H24	119.6
C7—C6—C5	120.1 (3)	C23—C24—H24	119.6
C7—C6—H6	120.0	C24—C25—C26	119.5 (4)
C5—C6—H6	120.0	C24—C25—H25	120.2
C6—C7—C8	120.5 (3)	C26—C25—H25	120.2
C6—C7—H7	119.7	C25—C26—C20	119.6 (3)
C8—C7—H7	119.7	C25—C26—H26	120.2
C7—C8—C3	119.2 (3)	C20—C26—H26	120.2
C7—C8—C9	121.9 (3)	C28—C27—C32	118.1 (3)
C3—C8—C9	118.9 (3)	C28—C27—C19	122.5 (3)
C10—C9—C8	122.2 (3)	C32—C27—C19	119.4 (3)
C10—C9—H9	118.9	C29—C28—C27	120.8 (3)
C8—C9—H9	118.9	C29—C28—H28	119.6
C9—C10—C1	121.3 (3)	C27—C28—H28	119.6
C9—C10—H10	119.4	C28—C29—C30	120.4 (3)
C1—C10—H10	119.4	C28—C29—H29	119.8
C12—C11—C16	120.8 (3)	C30—C29—H29	119.8
C12—C11—N2	120.2 (3)	C31—C30—C29	120.1 (3)
C16—C11—N2	119.0 (3)	C31—C30—H30	120.0
C13—C12—C11	117.6 (3)	C29—C30—H30	120.0
C13—C12—C17	119.2 (3)	C30—C31—C32	121.1 (3)
C11—C12—C17	123.0 (3)	C30—C31—H31	119.5
C14—C13—C12	121.4 (4)	C32—C31—H31	119.5
C14—C13—H13	119.3	C31—C32—C27	119.6 (3)
C12—C13—H13	119.3	C31—C32—C33	122.4 (3)
C15—C14—C13	120.8 (4)	C27—C32—C33	118.0 (3)
C15—C14—H14	119.6	C34—C33—C32	122.7 (3)
C13—C14—H14	119.6	C34—C33—H33	118.7
C14—C15—C16	119.5 (4)	C32—C33—H33	118.7
C14—C15—H15	120.3	C33—C34—C18	121.4 (3)
C16—C15—H15	120.3	C33—C34—H34	119.3
C15—C16—C11	119.9 (3)	C18—C34—H34	119.3
			
N4—Cu—O1—C1	−155.0 (2)	N2—C11—C12—C13	−179.8 (3)
N2—Cu—O1—C1	38.6 (2)	C16—C11—C12—C17	173.7 (3)
N4—Cu—O2—C18	−35.1 (2)	N2—C11—C12—C17	−5.4 (5)
N2—Cu—O2—C18	131.2 (2)	C11—C12—C13—C14	1.2 (5)
C2—N1—N2—C11	−169.5 (2)	C17—C12—C13—C14	−173.4 (4)
C2—N1—N2—Cu	15.6 (4)	C12—C13—C14—C15	0.3 (7)
O1—Cu—N2—N1	−35.7 (2)	C13—C14—C15—C16	−2.3 (7)
O2—Cu—N2—N1	145.4 (2)	C14—C15—C16—C11	2.7 (6)
N4—Cu—N2—N1	−107.3 (4)	C12—C11—C16—C15	−1.2 (5)
O1—Cu—N2—C11	150.0 (2)	N2—C11—C16—C15	177.9 (3)
O2—Cu—N2—C11	−28.9 (2)	Cu—O2—C18—C19	26.8 (4)
N4—Cu—N2—C11	78.4 (5)	Cu—O2—C18—C34	−153.5 (2)
C19—N3—N4—C20	−179.6 (2)	N4—N3—C19—C18	−11.1 (4)
C19—N3—N4—Cu	−11.9 (4)	N4—N3—C19—C27	172.2 (3)
O1—Cu—N4—N3	−150.1 (2)	O2—C18—C19—N3	3.2 (5)
O2—Cu—N4—N3	29.4 (2)	C34—C18—C19—N3	−176.5 (3)
N2—Cu—N4—N3	−78.9 (5)	O2—C18—C19—C27	179.7 (3)
O1—Cu—N4—C20	16.8 (2)	C34—C18—C19—C27	0.0 (4)
O2—Cu—N4—C20	−163.6 (2)	N3—N4—C20—C26	−125.9 (3)
N2—Cu—N4—C20	88.1 (4)	Cu—N4—C20—C26	65.3 (3)
Cu—O1—C1—C2	−23.7 (4)	N3—N4—C20—C21	59.0 (4)
Cu—O1—C1—C10	159.5 (2)	Cu—N4—C20—C21	−109.8 (3)
N2—N1—C2—C1	15.6 (4)	C26—C20—C21—C23	1.3 (5)
N2—N1—C2—C3	−172.6 (2)	N4—C20—C21—C23	176.2 (3)
O1—C1—C2—N1	−12.5 (5)	C26—C20—C21—C22	−176.5 (3)
C10—C1—C2—N1	164.3 (3)	N4—C20—C21—C22	−1.6 (5)
O1—C1—C2—C3	176.0 (3)	C20—C21—C23—C24	−0.4 (5)
C10—C1—C2—C3	−7.1 (4)	C22—C21—C23—C24	177.5 (4)
N1—C2—C3—C4	12.8 (4)	C21—C23—C24—C25	0.1 (6)
C1—C2—C3—C4	−175.0 (3)	C23—C24—C25—C26	−0.7 (6)
N1—C2—C3—C8	−168.9 (3)	C24—C25—C26—C20	1.6 (5)
C1—C2—C3—C8	3.3 (4)	C21—C20—C26—C25	−1.9 (5)
C8—C3—C4—C5	0.3 (5)	N4—C20—C26—C25	−177.0 (3)
C2—C3—C4—C5	178.6 (3)	N3—C19—C27—C28	−4.4 (4)
C3—C4—C5—C6	−1.1 (6)	C18—C19—C27—C28	178.7 (3)
C4—C5—C6—C7	0.7 (6)	N3—C19—C27—C32	176.0 (3)
C5—C6—C7—C8	0.3 (6)	C18—C19—C27—C32	−0.9 (4)
C6—C7—C8—C3	−1.0 (5)	C32—C27—C28—C29	−1.9 (5)
C6—C7—C8—C9	178.8 (3)	C19—C27—C28—C29	178.5 (3)
C4—C3—C8—C7	0.7 (4)	C27—C28—C29—C30	1.1 (5)
C2—C3—C8—C7	−177.7 (3)	C28—C29—C30—C31	0.2 (6)
C4—C3—C8—C9	−179.1 (3)	C29—C30—C31—C32	−0.6 (6)
C2—C3—C8—C9	2.5 (4)	C30—C31—C32—C27	−0.3 (5)
C7—C8—C9—C10	175.7 (3)	C30—C31—C32—C33	179.9 (3)
C3—C8—C9—C10	−4.5 (5)	C28—C27—C32—C31	1.5 (4)
C8—C9—C10—C1	0.5 (5)	C19—C27—C32—C31	−178.9 (3)
O1—C1—C10—C9	−177.7 (3)	C28—C27—C32—C33	−178.7 (3)
C2—C1—C10—C9	5.4 (5)	C19—C27—C32—C33	0.9 (4)
N1—N2—C11—C12	139.4 (3)	C31—C32—C33—C34	179.7 (3)
Cu—N2—C11—C12	−45.6 (4)	C27—C32—C33—C34	0.0 (5)
N1—N2—C11—C16	−39.7 (4)	C32—C33—C34—C18	−0.8 (5)
Cu—N2—C11—C16	135.3 (3)	O2—C18—C34—C33	−178.9 (3)
C16—C11—C12—C13	−0.7 (5)	C19—C18—C34—C33	0.8 (4)
